# Validation of the Cognitive-Emotional Perspective Taking test in patients with neurodegeneration

**DOI:** 10.1177/13872877251317683

**Published:** 2025-03-03

**Authors:** Jonathan Adrián Zegarra-Valdivia, Tal Shany-Ur, Myrthe Gwen Rijpma, Patrick Callahan, Pardis Poorzand, Scott Grossman, Bailey McEachen, Joel H Kramer, Bruce L Miller, Katherine P Rankin

**Affiliations:** 1 Global Brain Health Institute [GBHI], University of California San Francisco, San Francisco, CA, USA; 2 Achucarro Basque Center for Neuroscience, Leioa, Spain; 3 Universidad Señor de Sipán, Chiclayo, Perú; 4 Centro de Investigaciones Biomédicas en Red Enfermedades Neurodegenerativas (CIBERNED), Madrid, Spain; 5 Department of Neurology, Memory and Aging Center, University of California San Francisco, San Francisco, CA, USA

**Keywords:** Alzheimer's disease, frontotemporal degeneration, neurodegeneration, perspective taking, test validation, theory of mind

## Abstract

**Background:**

Theory of mind (ToM) is crucial for socioemotional interaction. ToM deficits may explain behavioral changes in dementia, especially Alzheimer's disease (AD) and frontotemporal dementia (FTD).

**Objective:**

This study examined the psychometrics of a new ToM test in healthy adults, identified ToM differences in dementia syndromes, and assessed if ToM scores predict neuropsychiatric function and real-life behavior.

**Methods:**

The UCSF Cognitive and Emotional Perspective Taking Test (CEPT) was evaluated in 195 healthy adults (age: 42.69 ± 16.20) and in a clinic cohort of 304 participants (age: 64.07 ± 9.2). Participants included healthy controls, AD, behavioral-variant frontotemporal dementia (bvFTD), semantic variant primary progressive aphasia (svPPA), non-fluent PPA (nfvPPA), and progressive supranuclear palsy (PSP) patients. CEPT's psychometrics were assessed, and ToM differences and predictions of neuropsychiatric symptoms were analyzed using regression models.

**Results:**

In controls, CEPT showed good validity and reliability. In patients, CEPT scores correlated with executive and emotional measures, but not language measures, showing good construct validity. Cognitive ToM was most impaired in AD and bvFTD, with less impairment in svPPA and PSP, and all patient groups showed impaired emotional ToM. ToM performance predicted real-life neuropsychiatric behavior, including anxiety, apathy, disinhibition, and aberrant motor behaviors.

**Conclusions:**

ToM deficits appear early in dementia syndromes and predict neuropsychiatric behavior. Assessing ToM and social cognition with ecologically valid tasks may help identify altered social cognition in early neurodegeneration.

## Introduction

Theory of mind (ToM) is a complex cognitive mechanism that involves predicting the content of other people's minds (including knowledge, emotions, beliefs, and intentions), often in circumstances where that content may differ from that in one's own mind.^1,2^ ToM ability develops early during infancy and seems to stabilize during early childhood, though the accuracy of ToM continues to evolve into adulthood with increasing socioemotional experience.^3,4^ By allowing accurate representation of others’ minds, this core cognitive process supports conventional socioemotional interaction in everyday life by facilitating the correct interpretation of others’ behavior and adaptation to changing social contexts.^5^

As a construct, ToM can be divided concerning whether the mental content being processed is cognitive or affective. Cognitive ToM can evaluate others’ knowledge, beliefs, or intentions, while emotional ToM involves understanding others’ moods and emotions.^6^ Both cognitive and emotional ToM abilities are predicted by attention and affective intelligence during adolescence, and there are sex differences in how cognitive processes such as working memory and language comprehension influence ToM.^7^ Additionally, both processes have unique but overlapping neural representations.^6,8^

Neuroimaging studies have demonstrated a ToM-related brain network that includes the medial prefrontal cortex, temporal poles, anterior and posterior midline regions, dorsal and ventral temporal-parietal junction (temporoparietal and supramarginal gyrus), and superior temporal gyrus.^9–11^ Multiple studies have found that attentional processes, mediated by the salience network, act as a gating mechanism controlling the activation of other cognitive networks that are needed to perform ToM effectively, such as set-shifting and memory.^12,13^

Research consistently reports ToM deficits in different clinical disorders, including schizophrenia, bipolar and unipolar depression, borderline personality disorder, anorexia, and neurodegenerative syndromes.^14–18^ In the latter case, understanding the effect of normal aging on ToM is crucial to elucidate how to assess it efficiently during early prodromal phases of neurodegeneration, during the normal-to-pathological aging transition. Existing literature reflects mixed findings in this area, with some showing no age-related difference in ToM abilities in older adults,^19,20^ but others indicating a decline in ToM abilities with aging, regardless of the task used.^21–23^

Many studies suggest ToM alterations commonly occur in Alzheimer's disease (AD), behavioral-variant frontotemporal dementia (bvFTD), dementia with Lewy bodies, and primary progressive aphasia syndromes (PPA).^12,18,24–30^ However, different imaging studies of ToM in patients with neurodegeneration tend to reveal inconsistent neural relationships, perhaps in part because there is no standardized test to assess ToM in this population,^31^ and tests often do not control for the deficits in non-ToM aspects of cognition that are common in these patients, such as memory, executive functioning, or emotion reading.

Furthermore, studies of ToM encounter problems because there is not a clear consensus regarding the theoretical framework or scope of the construct of ToM.^12^ Hence, research tasks used to study ToM differ across a number of dimensions, including (1) which mental states are considered to involve ToM (e.g., knowledge of visual or social perspective versus prediction of intention), (2) testing context and modality of stimuli (e.g., text-based, cartoon scenarios, and others), and (3) the goal or target for which ToM ability is implemented (e.g., understanding perspective versus selecting the most empathic behavioral response), which is particularly relevant to the overlap between ToM and other cognitive processes like emotional empathy and executive functioning.^11,32^ All of this testing diversity has further contributed to incongruence across MRI studies attempting to delineate the specific anatomic substrate of ToM in neurodegenerative disease patients and, more generally.^33^

Ideally, ToM tests designed for use with patients with cognitive deficits would disambiguate the elements of ToM being tested and would reduce or explicitly account for overlap between canonical ToM elements and contributions from other cognitive domains. Of course, to fractionate the construct of ToM will limit its ecological validity, i.e., how well the test reflects the capacity to employ holistic ToM reasoning in a realistically challenging setting, but this trade-off may be necessary to enhance psychometric rigor for face-to-face testing. For example, ToM tests for these patients should have stratified elements clearly delineating the executive contribution to patients’ ToM deficits (e.g., set-shifting, inhibitory control, and working memory for social material). This might include prioritizing the “perspective taking” elements of ToM in the task, would improve the psychometric precision for this one specific cognitive function that is not directly measured by other executive tasks. A task might also include trials that intentionally range decision-making difficulty from simpler to more complex executive demands. Similarly, patients with language deficits might have difficulty processing long blocks of text, or those with visuospatial impairments might be less able to decode line drawings. Thus, contextualization and multimodal presentation of each item, perhaps through video stimuli or with simultaneous audio narration of written text, will aid patients in focusing on and comprehending key elements of test items so that variance on the test corresponds more directly to their ability to perform perspective taking. Also, because perspective taking is conceptually distinct from emotion reading but may still depend on it,^12^ particularly when interpreting real-life situations, a task designed for patients with neurodegenerative disease might best dissociate emotional perspective taking (i.e., recognizing when not all individuals share the same information about other individuals’ emotional state) from pure emotion reading. In this way, a patient with focal deficits in face reading or emotion labeling who could still accurately dissociate perspectives could pass the test, and the clinical interpretation could be that their perspective taking is intact while their emotion reading is impaired. Thus, for patient evaluation, a test may be more precise if it incorporates a simplified, multimodal, but well-controlled approach that evaluates specific aspects of ToM processing, and uses realistic stimuli that distinguish cognitive from emotional elements where possible.

In accordance with these principles, our group at the Memory and Aging Center (MAC) at the University of California (UCSF) developed the Cognitive-Emotional Perspective Taking (CEPT) Test to identify focal impairments in cognitive (cToM) and emotional (eToM) perspective-taking in patients with neurocognitive disorders who show a wide range of non-ToM cognitive impairments in domains that could impact their test performance. Here, we describe the development and validation of the test, including whether the CEPT could detect early changes in social cognition in neurodegenerative conditions, and whether these changes corresponded to real-life social behavior. To achieve this, we first (1) identified the psychometric properties of the cToM and eToM components of the CEPT in a sample of healthy control adults, then (2) identified differences in CEPT performance across dementia syndromes, and finally (3) determined if CEPT performance predicted neuropsychiatric function and real-life social behavior as rated by an informant.

## Methods

### Sample

Participants were recruited from longitudinal observational studies at the UCSF MAC (San Francisco, CA, USA). We evaluated two different sub-samples: (1) a control cohort comprised of neurologically healthy individuals across the adult age span (*n = *195; mean age 42.69 ± 16.2, range 19–78), selected to show the general psychometric properties of the task including normative data, and (2) a clinic cohort (*n = *304) of individuals over 40 years old, (mean age 64.07 ± 9.2, range 46–87), selected to demonstrate the properties of the UCSF ToM task in aging adults with neurodegenerative disease, its intended population (41 neurologically healthy controls (NC); 34 with biomarker confirmed probable AD according to the McKhann criteria:^34^ 96 with bvFTD according to the ISFTD criteria;^35^ 38 with semantic variant primary progressive aphasia (svPPA) and 47 non-fluent PPA (nfvPPA) according to the ICFTD criteria:^36^ and 48 with progressive supranuclear palsy (PSP) according to the Höeglinger criteria.^37^

All patients also underwent a diagnostic evaluation, including complete clinical history, neurologic examination, neuropsychological evaluation, and functional/neuropsychiatric evaluation. AD patients were included only if they had confirmed amyloid pathology based on amyloid imaging or cerebrospinal fluid markers, as described in previous studies.^38,39^ The final diagnoses were made in a multi-disciplinary clinical consensus conference. All healthy controls were recruited from the community and underwent comprehensive neurologic evaluation, neuroimaging, and neuropsychological testing and were included only if they showed typical cognitive performance, did not have any neurologic, primary psychiatric, or significant medical illnesses, and had Clinical Dementia Rating (CDR^®^) score of zero.

### Procedures: Participant evaluation

Participants underwent direct assessment in a quiet room administered by neuropsychologically trained study personnel. Participants were monitored for signs of distress or fatigue, which would prompt discontinuation of testing. Each participant also identified a study partner who was able to report on their daily functioning via semi-structured interviews and questionnaires. Patients in the clinical cohort were evaluated with the whole test battery once; while all healthy controls were assessed at least once, a subset (*n = *60) underwent repeat testing with the cToM condition of the CEPT at two weeks and 1-year post-baseline to evaluate test-retest reliability and test stability.

### Ethical statement

Written informed consent was obtained from all participants in consultation with their caregivers, after full evaluation of their capacity to provide consent. Human procedures followed Helsinki guidelines and were approved by the University of California San Francisco Committee on Human Research.

### Direct socioemotional testing

#### Cognitive Emotional Perspective Taking (CEPT) test

The CEPT was developed to measure an examinee's ability to conduct the perspective-taking processes required to correctly infer others’ knowledge and beliefs based on watching narrated videos of characters interacting and answering structured questions. It contains two conditions measuring cognitive (cToM) and emotional (eToM) perspective-taking, with eight scenarios per condition (four “cheat” and four “no cheat”), and three questions per scenario. There is no sound directly from the video, but a voice-over narration is provided that describes the interaction on the screen. In the cToM condition, characters interact around an object, one character departs, and then that object is moved by the second character, who may or may not be unwittingly observed by the first character. The eToM condition follows the same structure, but instead of an object moving, the emotion of one character changes with or without the knowledge of the second character. To ensure any perspective-taking deficits are not conflated with deficits in emotion reading, the emotions of the characters are always explicitly named by the narrator; thus, the examinee does not need to be able to read emotions while performing the task, only to understand the perspective of one character on the other's named emotional state.

After each scenario, the examinee is shown a set of still photographs of key moments in the video to refresh their memory and focus their attention, below which they are asked three questions to assess (1) whether they understood and recall a fact about the video (control question: e.g., “Where is the bag?”/“What emotion is Ellen feeling now?”), (2) what one character thinks the second character knows or believes about the object (cToM) or emotion (eToM) (first order ToM question: e.g., “Where does Jack think the bag is now?”/“What does Jack think Ellen is feeling now?”), and (3) what one character thinks the second character thinks the first character believes about the object or emotion (second-order ToM question: e.g., “Where does Jack think Ellen thinks the bag is now?”/ “What does Ellen think Jack thinks Ellen is feeling now?”). Half of the scenarios involve a “cheat” condition, in which one of the characters is unwittingly observed by the other, making ToM deductions more executively complex. The maximum score for each condition is 8 points for the control question, and 16 total points for answering the first- and second-order ToM questions correctly.

This structure allows assessment of examinees’ ability to conduct perspective-taking in order to correctly identify characters’ knowledge and beliefs by making first- and second-order ToM inferences of graded executive complexity about non-emotional concrete and visually presented stimuli (cToM) and explicitly labeled emotional material, distinct from any concurrent emotion reading deficits (eToM). Performance on the control scale is designed to indicate whether the individual's other non-social cognitive deficits may have interfered with their ability to accurately perform the ToM elements of the task. Each condition takes approximately 10 min each to complete. The test videos, photos, and questions can be integrated into any standard questionnaire administration platform; for this study, the UCSF instance of the Qualtrics survey tool was used (http://www.qualtrics.com). While the test can be self-administered, for the purposes of this study a psychometrician was present to assist in case participants with cognitive disorders became confused or distracted while taking the task.

#### Emotion measures

We also administered face-to-face tests to evaluate participants’ capacity for emotional reading and mood. Patients also underwent testing with either of two video-based emotion reading tests over the multi-year time course of the study. The Awareness of Social Inference Test Emotion Evaluation Task (TASIT-EET) is designed for the clinical assessment of emotion reading based on videos of dynamic social interactions where basic emotions are expressed via semantically neutral scripts.^40^ For this study, we used the first 14 of the 28 TASIT-EET videos for brevity. After watching the ∼20-s videos, participants were asked to select one of the six basic emotions (happy, surprised, angry, frightened, disgusted, or sad) plus neutral. This test is commonly used for evaluation of persons with dementia, showing distinct performance profiles across syndromes and strong correspondence with underlying brain function.^41,42^ The Dynamic Affect Recognition Test (DART) is a similarly structured video-based test for which participants watch twelve short (∼20-s) videos of an actor portraying an emotion via a semantically neutral script, then, they are given the same six basic emotions (without neutral) as options to describe the emotion the character displayed in the video, for a maximum total score of 12. Participants over the long timespan of the study had either one or the other task, but because they had similar structure and psychometrics, we combined them to create an Emotion Reading summary score. We used the EET score when available or used raw DART*1.17 if EET was not available, thus yielding a total score of 14 for either test. With the assistance of the examiner, participants also completed the Geriatric Depression Scale (GDS) to self-identify symptoms of depression.^43^

### Direct neuropsychological testing

We use the Mini-Mental State Examination (MMSE) as a general measure of cognitive impairment. The neuropsychological evaluation used cognitive measures of each primary cognitive domain (memory, executive functioning, language, and visuospatial functioning). Details on the neuropsychological protocol can be found elsewhere;^15,44,45^ briefly, tests included the California Verbal Learning Test (Short form) for verbal memory, the Benson Figure for visuoconstruction and visual memory; Stroop color-naming speed and color-word interference speed, Trailmaking test speed, digit span forwards and backwards, and the Design Fluency Condition 1 task from the Delis-Kaplan Executive Function Scales to measure executive functioning; and the 15-item abbreviated version of the Boston Naming task, Animal fluency, D-word fluency, and the Wide Range Achievement Test reading condition to evaluate the language domain.

### Informant-based measures of real-life functioning

#### Behavior

To obtain an observer-based measure of participants’ real-life behavioral symptoms, family member informants were asked to report on participants via semi-structured interviews and questionnaires. We used the 12 subscale scores (frequency by severity product) and the total score from the Neuropsychiatric Inventory (NPI).^46^ We also used the Revised Self-Monitoring Scale (RSMS)^47^ as an informant-based measure of participants’ socioemotional sensitivity and responsiveness to subtle emotional expressions during face-to-face interactions.^48^

#### Disease severity

The CDR Sum of Boxes Score and Dementia Staging Instrument and the Global CDR score were recorded as the primary measures of disease severity.^49^

### Procedures: Statistical analyses

Statistical analysis was performed using IBM SPSS Statistics software (Armonk, NY, USA) and Graph Pad Prism 6 software (San Diego, CA, USA). Depending on the number of independent variables, degree of normality of data distribution (Kolmogorov–Smirnov normality test), and the experimental groups compared, we used Student's t-test or two-way ANOVA followed by Dunnett's multiple comparison test. For non-normally-distributed data, we used the Mann–Whitney U test to compare two groups or a non-parametric alternative to multi-factorial ANCOVA (Quade's test)^50^ with a post hoc analysis, using Dunnett's multiple comparison test. For Spearman's correlations, the following interpretations were used: 0.0–0.25 “very weak,” 0.26–50 “weak,” 0.51–0.75 “moderate to strong,” 0.76–1.0 “very strong to perfect”.^51^

### Evaluation of CEPT psychometric properties

To evaluate the content validity of the CEPT, we used Cronbach alpha, intraclass correlation coefficients, and subscale-test correlation approaches to assess the homogeneity and internal consistency of the CEPT in the control cohort. The Bland-Altman was then used to plot the test-retest reliability of cToM total scores in the control cohort at two-time intervals (2 weeks and one year after the first evaluation), reflecting the test stability in a healthy aging cohort. This graphical method offers a means to assess the concordance between repeated measurements. It involves plotting the disparities between test and retest scores against each participant's average test and retest scores. Confidence intervals are computed for the mean difference to ascertain whether it significantly departs from zero.^52^ Means and standard deviations of all CEPT subscale scores were derived in the control cohort to provide normative data for clinical evaluation.

To examine the convergent and discriminant validity of the CEPT, i.e., the degree to which scores reflect predicted associations with other domains of function outside of Theory of Mind, we used Spearman's rank correlation coefficient, performed in each group (control cohort, clinic, and total). We expected higher degrees of association between CEPT scores and (1) disease severity (as measured by CDR^®^), (2) level of executive cognitive control (represented by Stroop interference score), and (3) level of socioemotional functioning (represented by Emotion Reading score). We also examined the association with (4) speech and language functions (represented by phrase repetition and verbal fluency scores) to show the discriminant validity of the CEPT, predicting they should have lower associations. Because the CEPT data was not distributed normally, concurrent validity was assessed by determining Spearman's rank correlation coefficient (*ρ*) to measure the strength of a monotonic relationship between the CEPT and each test score.

For criterion-related validity, i.e., the capacity of the CEPT to predict diagnostic group membership, we compared all CEPT summary scores from both cToM and eToM conditions across all groups (NC, AD, bvFTD, svPPA, nfvPPA, and PSP) using non-parametric ANCOVA, with the expectation that poorer scores on the CEPT would predict patient group membership and that syndromes with known behavioral disorders such as bvFTD and svPPA would show worse performance than those with non-behavioral syndromes such as AD and nfvPPA.

### Additional analyses

Multiple regression modeling examined the relationship between CEPT performance and other cognitive functions in control and combined samples, controlling for age and sex. Initially, both forward and backward stepwise analyses were conducted for predictor validation utilizing all the assessed cognitive scores. Subsequently, the variables that remained significant via both methods were selected for final modeling to ascertain the stability of the models. A conclusive model was developed using the “entry method,” with 95% bias-corrected bootstrapped confidence intervals involving 10,000 repetitions (to address non-normally distributed data).

Finally, we analyzed CEPT scores to determine if they predict real-life behaviors reported by informants (i.e., neuropsychiatric symptom burden according to the NPI and poor interpersonal sensitivity according to the RSMS). We performed linear regressions with bootstrapping. We also controlled for bvFTD group membership because bvFTD patients are known to have significantly worse neurobehavioral symptoms, and we wished to identify any relationship between CEPT and behavior that generalized beyond individuals with bvFTD.

## Results

### Sample characteristics

*Control cohort.* In the neurologically healthy cohort, aged 19 to 78, we did not find a significant influence of age or education on CEPT scores. In the cToM condition, females performed slightly better on the Control Task Total score (*M = *3.97 versus 3.87 in males; *U:* 4342.5, *p < *0.05) and some eToM tasks (Table 1), though the magnitude of the score differences was so small they would be unlikely to have clinical relevance on an individual level. Data for normative CEPT performance clustered by age (≤45, 46–70, 71≤) can be also found in Table 1.

**Table 1.** Demographic characteristics and CEPT performance in the normal control sample grouped by age and sex.

Control cohort		Younger than 45		Between 46–70		Older than 70		Statistic	Male		Female		Statistic
		Mean	SEM	Mean	SEM	Mean	SEM	*p*	Mean	SEM	Mean	SEM	*p*
Sample w/cToM Demographics	N	67		86		42			84		111		
	Sex (M/F) ^a^	29/38		32/54		23/19		0.17					
	Age	33	0.86	62.72	0.716	77.33	0.833	0.001	56.37	2.06	55.12	1.74	0.389
	Education ^b^	16.39	0.398	17.32	0.218	17.95	0.426	0.009	17.52	0.296	16.87	0.252	0.062
Sample w/eToM Demographics	N	56		27		11			40		54		
	Sex (M/F) ^a^	22/34		08/19*		10/01*		0.002					
	Age	32	0.95	58.3	1.37	77.4	1.42	0.001	48.62	3.271	42.13	2.087	0.155
	Education ^b^	16.2	0.44	17.6	0.40	17.5	0.78	0.816	16.97	0.451	16.65	0.416	0.651
*Cognitive Theory of Mind*													
Control Task	No Cheat	3.96	0.025	3.99	0.012	3.95	0.033	0.424	3.96	0.02	3.97	0.015	0.729
	Cheat	3.97	0.021	3.99	0.012	3.95	0.033	0.495	3.92	0.035	3.93	0.025	0.995
	Total	7.93	0.039	7.98	0.016	7.9	0.046	0.242	3.87	0.056	3.97	0.015	0.041
First Order Task	No Cheat	3.93	0.039	3.94	0.025	3.88	0.051	0.608	3.96	0.02	3.98	0.013	0.44
	Cheat	3.94	0.036	3.91	0.032	3.79	0.064	0.613	3.85	0.043	3.93	0.025	0.103
	Total	7.87	0.06	7.85	0.039	7.67	0.081	0.596	3.87	0.041	3.91	0.03	0.379
Second Order Task	No Cheat	3.9	0.038	3.95	0.047	3.93	0.04	0.153	7.93	0.033	7.95	0.02	0.639
	Cheat	3.82	0.056	3.93	0.028	3.93	0.04	0.754	7.76	0.058	7.86	0.036	0.201
	Total	7.72	0.076	7.88	0.063	7.86	0.064	0.251	7.74	0.081	7.88	0.036	0.151
Total Scores	Total No Cheat	11.78	0.082	11.88	0.053	11.76	0.095	0.362	11.75	0.081	11.87	0.041	0.314
	Total Cheat	11.73	0.081	11.83	0.05	11.67	0.088	0.251	11.68	0.074	11.82	0.043	0.123
	Total Correct cToM	15.58	0.119	15.73	0.075	15.52	0.114	0.327	15.5	0.112	15.74	0.055	0.095
	Total cToM	23.51	0.137	23.71	0.077	23.43	0.133	0.173	23.43	0.125	23.69	0.062	0.106
*Emotional Theory of Mind*													
Control Task	No Cheat	3.9	0.03	3.9	0.04	4	0	0.84	3.95	0.035	3.96	0.026	0.759
	Cheat	4	0	4	0	4	0	1	4	0	4	0	1
	Total	3.8	0.06	3.5	0.15	3.6	0.20	0.177	3.53	0.107	3.83	0.069	0.002
First Order Task	No Cheat	4	0	3.9	0.04	4	0	0.627	4	0	3.98	0.019	0.389
	Cheat	3.9	0.04	4	0	3.9	0.09	0.427	3.92	0.055	4	0	0.099
	Total	3.7	0.07	3.2	0.21	2.5	0.28	0.279	3.3	0.144	3.61	0.11	0.044
Second Order Task	No Cheat	7.9	0.03	7.9	0.05	8	0	0.688	7.95	0.035	7.94	0.031	0.906
	Cheat	7.9	0.04	8	0.01	7.9	0.09	0.427	7.93	0.055	8	0	0.099
	Total	7.5	0.10	6.7	0.24	6.1	0.32	0.742	6.83	0.175	7.44	0.129	0.001
Total Scores	Total No Cheat	11.7	0.07	11.4	0.17	11.6	0.20	0.231	11.48	0.119	11.8	0.081	0.004
	Total Cheat	11.7	0.09	11.1	0.22	10.4	0.34	0.282	11.23	0.174	11.59	0.117	0.043
	Total Correct eToM	15.5	0.11	14.7	0.24	14.1	0.37	0.736	14.75	0.202	15.44	0.129	0.001
	Total eToM	23.5	0.12	22.6	0.26	22.1	0.37	0.797	22.7	0.203	23.39	0.141	0.002

*a = χ*^2^, *b = *Kruskal-Wallis's test; *#p < *0.10; *p < 0.05; **p < 0.01; and ***p < 0.001.

#### Clinic cohort

The subset of 41 healthy controls that were age-matched to our neurodegeneration patients (age 40+) were compared to our patient groups by age, sex, and education and were found to be somewhat younger (*F:* 18.925, *p < *0.01), though education did not statistically differ. Sex distribution significantly differed across NC and patient groups (*F:* 62.348, *p < *0.001). Age and sex were therefore included as confounding covariates in subsequent comparative and regression analyses. Predictably, clinical test scores were significantly worse in the patient groups than the NCs, including the MMSE, Global CDR^®^ score, and CDR^®^ Sum of Boxes score (*p < *0.001). However, this cohort was generally very early in their disease progression (CDR^®^: 0.80 ± 0.53), which is optimal for demonstrating focal cognitive deficits across individuals, since with at more advanced disease stages, patients with neurodegenerative diseases will fairly uniformly fail executively demanding tasks.

### Psychometric properties of the CEPT

The primary psychometric characteristics of the CEPT were first evaluated in the control cohort. Additional reliability and construct validity analyses were then performed within the clinic cohort.

#### Reliability (control cohort)

Cronbach's alpha for cToM Total score (*α = *0.847) and eToM Total Score (*α = *0.813) reflected a good internal consistency^53^ (see Supplemental Table 1). Additional information about internal consistency in the clinic cohort, with alphas ranging from 0.91–0.94, can be found in Supplemental Table 2, and correlations between each subscale and the overall test for the control cohort can be found in Supplemental Table 3.

The retest reliability of the cToM Total score was evaluated using the Bland-Altman test (Supplemental Figure 1). The mean difference between test and retest responses across control participants after two weeks (0.08 ± 0.69; 95% *CI:* −1.28 to 1.45) indicated no significant change in results, showing good test-retest reliability. Similarly, no significant difference was found between baseline and one year (0.07 ± 0.09; 95% *CI:* −1.24 to 1.38), suggesting good test stability in healthy older adults.

#### Concurrent validity

Control cohort: In this sample, cToM and eToM Total scores were not significantly correlated. We analyzed convergent and divergent validity for cToM Total and eToM Total scores against Stroop (executive) and Emotion Reading (socioemotional) scores. We found that the CEPT scores did not significantly correlate with the executive or socioemotional domains. The lack of correlation among these measures reflects some ceiling effects and a lack of variance in performance in the control cohort. Clinical cohort: The clinical group, in contrast, did show a significant correlation between the cToM and eToM Total scores (*r = *0.646, *p < *0.001) (Supplemental Table 2). A moderate negative correlation was found between CDR^®^ and both cToM (*r = *−0.424, *p < *0.001) and eToM (*r = *−0.447, *p < *0.001), suggesting that poorer CEPT scores correspond with increasing clinical symptom severity. Significant, moderate correlations were found between the Stroop score and both cToM (*r = *0.441, *p < *0.001) and eToM Total scores (*r = *0.304, *p < *0.001), as well as between the Emotion Reading score and both cToM Total (*r = *0.413, *p < *0.001) and eToM Total scores (*r = *0.440, *p < *0.001). Thus, in a clinical sample, performance on the CEPT is distinct from, but related to, performance on executive and socio-emotional measures.

### Clinical correlates of the CEPT in neurodegenerative disease patients

#### Differential performance across patient groups

**Figure 1.**
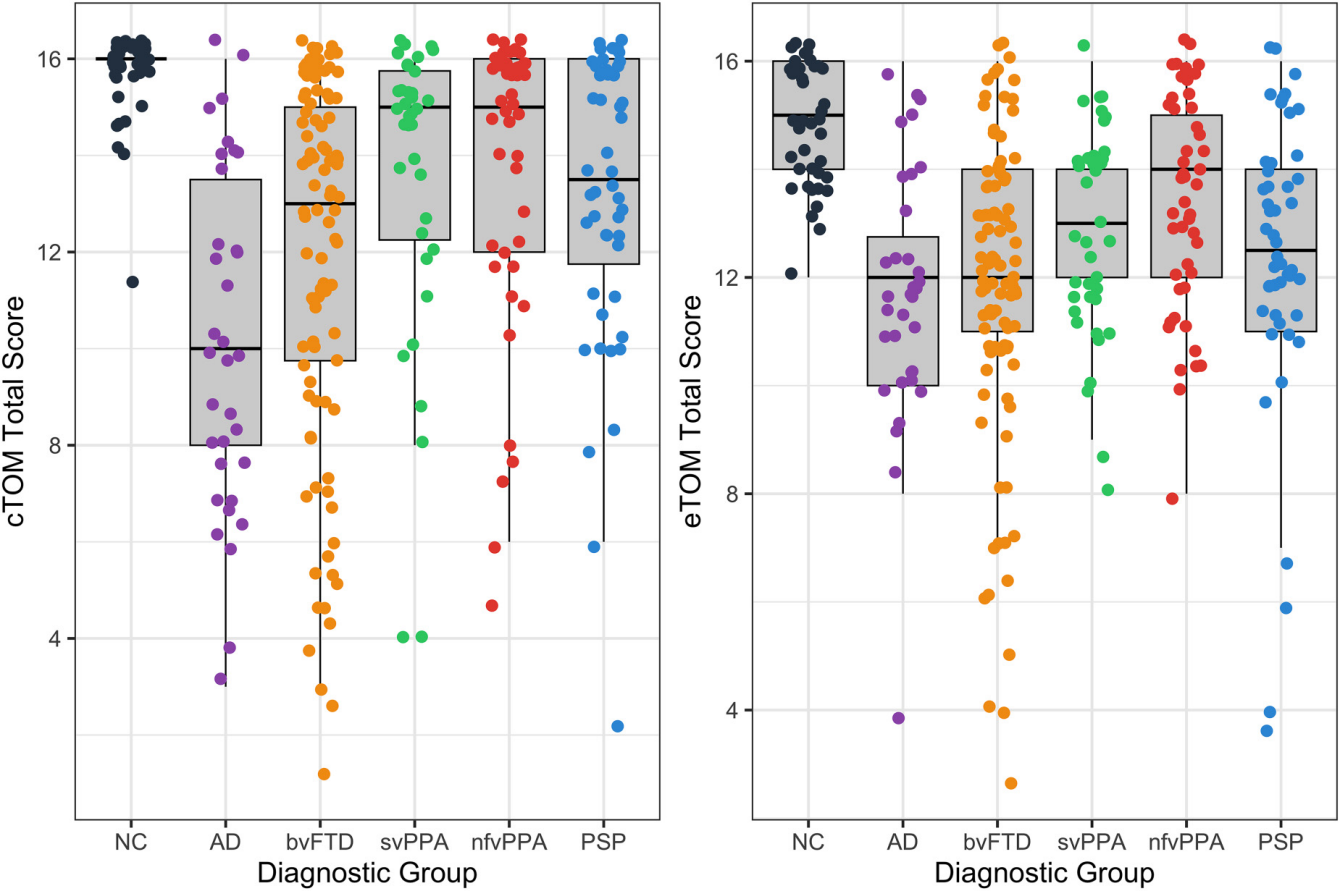
CEPT performance, including cognitive and emotional theory of mind conditions, across neurodegenerative disease syndrome groups. (a) cToM Total ToM score (out of 16); (b) eToM Total ToM score (out of 16).

The comparisons between NC and the clinical groups suggest good criterion validity for predicting neurodegenerative group membership using the CEPT (see Table 2). AD and bvFTD groups performed significantly worse than NCs for all the CEPT scores (*p < *0.001), scored quantitatively worse than other patient groups, and were more likely to fail the Control condition, suggesting a significant influence of general cognitive dysfunction on their CEPT theory of mind scores. svPPA and PSP scored better than AD and bvFTD patients and did not show impairment on the Control condition but still performed significantly worse than controls on both cToM Total score (*p < *0.01) and eToM Total score (*p < *0.001). nfvPPA patients performed usually on the cToM condition but scored significantly worse than controls on eToM Total scores (*p < *0.05). cToM and eToM distribution between clinic groups is in Figure 1.

**Table 2.** Demographic characteristics and CEPT performance in the clinical cohort by diagnostic group.

Clinic cohort		NC (*n = *41)		AD (*n = *34)		bvFTD (*n = *96)		svPPA (*n = *38)		nfvPPA (*n = *47)		PSP (*n = *48)		Statistics	
		Mean	SEM	Mean	SEM	Mean	SEM	Mean	SEM	Mean	SEM	Mean	SEM	*p*	*ηp*2
Demographics	Sex (M/F) ^a^	18/23		17/17		60/36		17/21		14/33		29/19		0.002	
	Age	56.49	1.79	65.76	1.551	63.82	0.822	65.45	1.129	67.68	1.165	69.19	1.073	0.001	0.477
	Education ^b^	17.31	0.384	16.76	0.42	16.5	0.329	16.89	0.448	16.2	0.475	16.49	0.543	0.936	0.004
Clinical Features	Total MMSE	28.8	0.206	22.39**	1.114	24.35**	0.486	24.94**	0.552	25.49**	0.726	24.98**	0.542	0.001	0.196
	Total CDR	0.076	0.0317	0.758**	0.0846	1.065**	0.0569	0.711**	0.0643	0.333**	0.0449	0.867**	0.0735	0.001	0.441
	CDR box score	0.152	0.0737	4.212**	0.4718	5.989**	0.3008	3.724**	0.3388	1.344**	0.2077	5.022**	0.38	0.001	0.504
*Cognitive Theory of Mind*															
Control Task	No Cheat	3.98	0.024	3.18**	0.229	3.52**	0.098	3.74	0.117	3.89	0.055	3.77	0.068	0.001	0.063
	Cheat	3.95	0.034	2.79**	0.226	3.09**	0.127	3.66	0.132	3.7	0.091	3.21*	0.168	0.001	0.118
	Total	3.88	0.1	2.76**	0.211	3.03**	0.135	3.32*	0.185	3.43	0.16	3.4	0.142	0.001	0.101
First Order Task	No Cheat	3.95	0.034	3**	0.198	3.64*	0.079	3.79	0.12	3.81	0.078	3.71	0.089	0.001	0.113
	Cheat	3.95	0.034	2.03**	0.237	2.89**	0.133	3.16*	0.198	3.49	0.125	3.15*	0.158	0.001	0.177
	Total	3.88	0.052	2.56**	0.194	2.86**	0.135	3.47	0.15	3.3	0.166	3.42	0.136	0.001	0.127
Second Order Task	No Cheat	7.93	0.041	6.18**	0.388	7.16*	0.158	7.53	0.222	7.7	0.113	7.48#	0.115	0.001	0.09
	Cheat	7.9	0.047	4.82**	0.372	5.98**	0.202	6.82*	0.247	7.19	0.174	6.35**	0.239	0.001	0.214
	Total	7.76	0.134	5.32**	0.324	5.9**	0.247	6.79#	0.307	6.72	0.304	6.81	0.245	0.001	0.145
Total Scores	Total No Cheat	11.8	0.106	8.74**	0.544	9.65**	0.296	10.71*	0.329	11.02	0.24	10.38*	0.302	0.001	0.146
	Total Cheat	11.78	0.074	7.59**	0.449	9.39**	0.26	10.42*	0.345	10.6	0.302	10.27#	0.313	0.001	0.221
	Total Correct cToM	15.66	0.142	10.15**	0.597	11.88**	0.398	13.61*	0.508	13.91	0.441	13.17*	0.445	0.001	0.224
	Total cToM	23.59	0.148	16.32**	0.897	19.03**	0.505	21.13*	0.618	21.62	0.511	20.65*	0.536	0.001	0.221
*Emotional Theory of Mind*															
Control Task	No Cheat	3.95	0.034	3.82	0.107	3.67*	0.078	3.79*	0.067	3.81	0.072	3.73*	0.077	0.001	0.048
	Cheat	4	0	3.82	0.079	3.57**	0.078	3.76	0.096	3.94	0.036	3.58*	0.126	0.001	0.092
	Total	3.61	0.115	2.44**	0.224	2.53**	0.142	2.55**	0.167	3	0.18	2.83	0.177	0.001	0.101
First Order Task	No Cheat	3.98	0.024	3.71	0.116	3.66*	0.074	3.53**	0.135	3.79	0.08	3.77	0.085	0.001	0.059
	Cheat	3.98	0.024	3.44*	0.153	3.4**	0.11	3.74	0.072	3.7	0.08	3.65	0.131	0.001	0.063
	Total	3.29	0.136	1.94**	0.227	2.28**	0.141	2.74	0.198	2.72	0.196	2.25*	0.187	0.001	0.091
Second Order Task	No Cheat	7.93	0.041	7.53	0.195	7.32*	0.137	7.32**	0.16	7.6#	0.124	7.5*	0.136	0.001	0.062
	Cheat	7.98	0.024	7.26*	0.204	6.97**	0.17	7.5*	0.105	7.64	0.098	7.23#	0.239	0.001	0.094
	Total	6.9	0.163	4.38**	0.277	4.81**	0.175	5.29**	0.238	5.72*	0.252	5.08**	0.226	0.001	0.193
Total Scores	Total No Cheat	11.56	0.131	10.09**	0.275	9.77**	0.24	10.11**	0.229	10.74#	0.205	10.15*	0.274	0.001	0.132
	Total Cheat	11.24	0.155	9.09**	0.357	9.33**	0.232	10*	0.28	10.21*	0.254	9.67*	0.288	0.001	0.127
	Total Correct eToM	14.88	0.172	11.65**	0.409	11.78**	0.295	12.79**	0.302	13.36*	0.302	12.31**	0.388	0.001	0.206
	Total eToM	22.8	0.186	19.18**	0.535	19.1**	0.398	20.11**	0.399	20.96*	0.374	19.81**	0.485	0.001	0.206

*a = χ*^2^, *b = *Kruskal-Wallis's test; *#p < *0.10; *p < 0.05; **p < 0.01; and ***p < 0.001.

#### Influence of cognition on CEPT performance

We used regression modeling to study the relationship between domain-specific cognitive deficits and CEPT performance in our patients with neurocognitive disorders (see Supplemental Table 4). We found that Emotion Reading, Stroop Interference (i.e., cognitive control), Benson Figure Copy (visuospatial functioning), and Benson Figure Delay (visuospatial memory) all contributed independently to the prediction of cToM performance in the clinical cohort (*p < *0.0001). eToM performance was predicted by Emotion Reading, Benson Figure Copy, California Verbal Learning Trials 1–4 Correct (auditory attention), and Design Fluency score (generation of nonverbal material) (*p < *0.0001).

#### CEPT as a predictor of real-life behavior

We analyzed the relationship between cToM Total score and behavior using models controlling for age, sex, and bvFTD group membership (Table 3). In the case of NPI subscales, cToM predicted Apathy and Disinhibition scores with a medium-size effect,^54^ with adjusted *R*^2^ of 26% and 20%, respectively, and showed a very weak prediction of anxiety (adjusted *R*^2^* = *3%). cToM Total score statistically significantly predicted RSMS socioemotional sensitivity score with an adjusted *R*^2^* = *28%, a medium-size effect according to Cohen.^54^

**Table 3.** Regression models analyzing real-life behaviors (NPI subscales and RSMS) predicted by cToM in the clinical cohort.

	B	95% CI		Bootstrap	SE B	*β*	*R* ^2^	*ΔR* ^2^
		LL	UL					
RSMS – Model							0.276	0.263**
Constant	28.401**	15.691	42.068	0.116	6.739			
Age	−0.027	−0.203	0.156	0.001	0.091	−0.017		
Sex	1.755	−1.378	4.806	−0.053	1.559	0.066		
bvFTD group	−13.209**	−16.25	−10.166	−0.014	1.549	0.121**		
cToM	0.433*	0.024	0.814	−0.005	0.201	−0.48*		
Anxiety – Model							0.04	0.025*
Constant	3.51	0.089	6.953	−0.01	1.739			
Age	−0.04	−0.083	0.003	0.001	0.022	−0.11		
Sex	−0.196	−0.881	0.507	0.001	0.352	−0.034		
bvFTD group	0.49	−0.298	1.333	0.009	0.414	0.145		
cToM	0.115*	0.027	0.196	0.001	0.043	0.082*		
Apathy – Model							0.26	0.248**
Constant	6.795**	2.178	11.314	−0.021	2.306			
Age	0.015	−0.048	0.079	0.001	0.032	0.027		
Sex	−1.325*	−2.271	−0.371	0.002	0.489	−0.155*		
bvFTD group	3.884**	2.873	4.902	−0.002	0.516	−0.127**		
cToM	−0.151*	−0.267	−0.029	0.001	0.061	0.436*		
Disinhibition – Model							0.207	0.194**
Constant	4.925*	0.514	9.184	−0.02	2.191			
Age	0.024	−0.031	0.078	0.001	0.028	0.048		
Sex	−0.552	−1.406	0.33	0.009	0.448	−0.07		
bvFTD group	3.088**	2.11	4.063	−0.003	0.5	0.376**		
cToM	−0.216*	−0.337	−0.086	0.002	0.064	−0.199*		

*^#^p < *0.10; *p < 0.05; **p < 0.001; NPI: Neuropsychiatric Inventory, RSMS: Revised Self-Monitoring Scale Total Score; cToM: Cognitive Theory of Mind scale; eToM: Emotional Theory of Mind scale; bvFTD: behavioral variant frontotemporal dementia syndrome.

Performing the same analysis with eToM Total score and behavior results (Table 4) showed that eToM significantly predicted several NPI neurobehavior scores, including NPI Total score (adj *R*^2^* = *24.5%), apathy (adj *R*^2^* = *28.3%), disinhibition (adj *R*^2^* = *19.3%, Aberrant Motor Behavior (adj *R*^2^* = *19.3%), and Aberrant Eating Behavior (adj *R*^2^* = *24.9%). eToM predicted RSMS score (adj *R*^2^* = *27.6%) at a medium-size effect, according to Cohen,^54^ even after controlling for bvFTD group membership.

**Table 4.** Multiple regression model analyzing real-life behaviors (NPI subscales and RSMS) predicted by eToM in the clinical cohort.

	B	95% CI		Bootstrap	SE B	*β*	*R* ^2^	*ΔR* ^2^
		LL	UL					
RSMS – Model							0.291	0.278**
Constant	21.365**	7.671	35.965	−0.004	7.194			
Age	−0.004	−0.183	0.169	0.001	0.09	−0.003		
Sex	1.664	−1.369	4.691	0.012	1.536	0.062		
bvFTD group	−12.658**	−15.666	−9.66	−0.008	1.517	0.174**		
eToM	0.89**	0.367	1.424	0.001	0.269	−0.46**		
*NPI-Q total scores and subscales*								
Total NPI – Model							0.258	0.245**
Constant	0.0001**	19.414	68.788	−0.081	12.55			
Age	−6.837	−0.314	0.316	0.001	0.162	0.001		
Sex	20.109*	−11.78	−1.913	0.008	2.499	−0.154**		
bvFTD group	−0.87*	14.791	25.602	0.01	2.757	−0.102*		
eToM	−0.87**	−1.697	−0.018	0.004	0.43	0.434***		
Apathy – Model							0.295	0.283**
Constant	9.963**	5.46	14.427	0.008	2.285			
Age	0.004	−0.055	0.062	6.624E-06	0.03	0.007		
Sex	−1.272*	−2.219	−0.317	0.004	0.486	−0.0148*		
bvFTD group	3.742**	2.765	4.74	0.006	0.508	−0.217**		
eToM	−0.355**	−0.514	−0.196	−0.001	0.081	0.419**		
Disinhibition – Model							0.203	0.19**
Constant	5.913*	1.757	10.144	−0.013	2.136			
Age	0.018	−0.034	0.07	0.001	0.027	0.038		
Sex	−0.494	−1.384	0.397	0.006	0.452	−0.063		
bvFTD group	3.056**	2.087	4.018	0.001	0.493	−0.187**		
eToM	−0.28**	−0.439	−0.115	−0.00006038	0.081	0.374**		
Motor – Model							0.206	0.193**
Constant	9.145**	4.513	13.681	9.145	0.018			
Age	−0.056*	−0.11	−0.003	−0.056	0	−0.111		
Sex	−0.356	−1.302	0.587	−0.356	0.006	−0.043		
bvFTD group	3.138**	2.078	4.206	3.138	−0.006	−0.132		
eToM	−0.206**	−0.377	−0.022	−0.206	−0.001	0.367		
Eating – Model							0.261	0.249**
Constant	8.85**	4.171	13.095	−0.07	2.295			
Age	−0.036	−0.089	0.019	0.001	0.027	−0.072		
Sex	−1.002*	−1.908	−0.089	0.009	0.461	−0.124*		
bvFTD group	3.565**	2.531	4.548	−0.001	0.515	0.424**		
eToM	−0.172*	−0.338	0.008	0.003	0.088	−0.112*		

*^#^p < *0.10; *p < 0.05; **p < 0.001; NPI: Neuropsychiatric Inventory, RSMS: Revised Self-Monitoring Scale Total Score; cToM: Cognitive Theory of Mind scale; eToM: Emotional Theory of Mind scale; bvFTD: behavioral variant frontotemporal dementia syndrome.

## Discussion

Our validation of the CEPT task for individuals with neurocognitive disorders showed that the CEPT has good internal consistency, 2-week test-retest reliability, and one-year test stability in a neurologically healthy older adult sample. The CEPT was particularly sensitive to ToM deficits in clinical populations for whom ToM is known to be significantly impaired, i.e., bvFTD and AD.^55,56^

The cognitive condition of the CEPT (cToM) showed a moderate correlation with tests of cognitive control, visuospatial processing, and socioemotional functioning and predicted real-life apathy, disinhibition, and socioemotional sensitivity. Performance on the emotional perspective-taking condition (eToM), intentionally designed to avoid any demand that the examinee infer or identify implicit emotions, also correlated with auditory attention and predicted real-life neuropsychiatric behaviors, including disinhibition and socioemotional sensitivity. Thus, the CEPT may be useful for measuring how the ability to predict others’ beliefs and intentions becomes altered in individuals with neurodegenerative conditions.

### Theory of mind in healthy aging

When the CEPT was used to examine cognitive and emotional perspective-taking comprehensively in our neurologically healthy control sample, using subscales representing tasks of increasing executive complexity (i.e., first and second-order ToM deliberations, with and without a cheat condition), we saw no effect of age on performance, with young-, middle-, and older-adults performing equally well.^57^ These results support the stability over the adult lifespan of the cognitive processes involved in understanding others’ beliefs and intentions and the robustness of socioemotional functioning even in late adulthood.^19,20,55^

Our study did not find differences in cognitive and emotional ToM capacity in healthy individuals, at least as measured by the CEPT test. Furthermore, we found no correlation between CEPT scores and other cognitive processes in our neurologically healthy sample, such as inhibitory control and emotion reading, though these correlations were found in our clinical sample. Potentially, the lack of results reflecting age-related nuances in ToM functioning in our study may have been because our healthy cohort showed some ceiling effects in their performance of the CEPT. This result contrasts with other reports, where investigators found ToM alterations associated with aging.^21,23^ This discrepancy may derive from multiple factors, such as differences in the measurement tasks across studies^58^ (e.g., written stories, pictures, or videos as stimuli; asking participants to show comprehension of narrative histories versus asking them to perform a social events analysis; measuring first- or second-order false beliefs in contrast to higher-order ToM). The degree to which a task is considered to be the speed of response and accuracy may also have resulted in some studies showing age-related ToM alterations.^23^ Aging and brain health are not uniform; thus, any other task differences that placed particular demands on the aspects of executive function that show greater individual differences and instability with age (e.g., working memory, information processing speed, inhibitory control) could result in age-related ToM differences, though it remains unclear the degree to which these findings truly reflect healthy participants’ ability to maintain ToM as they age.^59,60^

Our control participants were carefully selected and underwent a much more rigorous inclusion screening than is typically performed in studies of ToM in community-based samples, including comprehensive screening for neurologic disease, neuropsychological testing, neurologic examination, and structural MRI scanning. Arguably, this may have resulted in an unusually healthy control sample that does not genuinely reflect the full range of aging-related neurologic problems seen in community samples; however, it also suggests that ToM declines, and by extension declines in executive function and socioemotional processing, may reflect subclinical neurologic disease, and may not be a part of genuinely healthy aging.

### Impact of neurodegeneration on the theory of mind

Testing the ToM in individuals with concurrent cognitive deficits has always been challenging practically, both in clinical and research settings. Decoding others’ thoughts and intentions is a higher-order cognitive process that depends on numerous other subordinate cognitive functions, including memory, executive processing, and the ability to read emotions and social cues. Performance on ToM tests will also rely on the examinee's capacity to perform perceptual processing of the stimuli, whether presented in verbal or pictorial format. Thus, studies in clinical samples have repeatedly found correlations between ToM performance and numerous other cognitive domains, and our study further confirms this relationship. We found that in the individuals with neurodegenerative disease in our study, CEPT scores were strongly associated with inhibitory control, visual-constructional ability, auditory attention, visual memory, and the ability to spontaneously generate non-verbal material, suggesting an involvement of a wide range of circuits throughout the brain. However, this also confirms that when patients with neurodegeneration and other neurologic diseases have deficits in non-social cognitive domains, particularly in executive functioning, this not only impacts test performance but likely directly alters their capacity for cognitive and emotional perspective taking in real life.^60^

Many existing emotional ToM tasks require that emotion be inferred from the stimuli, either from facial or vocal expressions or contextual cues. The eToM condition of the CEPT was intentionally designed to dissociate the “perspective-taking” elements of ToM from pure emotion reading, instead explicitly telling examinees what emotions the actors were feeling but asking them to recognize when one character had never observed the emotion and to identify the limitations of their knowledge. This allows the eToM task to strongly parallel the cToM condition structurally but also makes it one of the first ToM tasks designed specifically for individuals with diverse patterns of cognitive deficits. Individuals with emotion reading impairments but no additional perspective-taking impairments should typically perform this task; conversely, an individual with an actual perspective-taking impairment will show deficits on the CEPT, even if their emotion reading remains intact.

Many brain regions focally disrupted by neurodegeneration seem to be required for ToM reasoning. There is a growing consensus among researchers that a widely distributed set of brain regions are involved in ToM, including crucial nodes in the medial prefrontal cortex, the temporal-parietal junction, the posterior cingulate cortex, the medial orbitofrontal cortex, and the precuneus.^61,62^ In addition to this core “mentalizing network,” other brain networks give support to ToM processing, including the salience network (involved in detecting salient stimuli and allocating attention to those stimuli, focally affected in bvFTD^63^ and default mode network (involved in memory processes, imagination, and scenario prediction, focally affected in AD^64,65^ For example, there is evidence that the mentalizing network may rely on the salience network to detect social cues, and the default mode network may provide key personal memories for building inferences about others’ intentions.^12,66,67^ Developing a more nuanced understanding of the neural basis of ToM will deepen not only our knowledge of how this ability develops in childhood but also how it can become impaired in neurodegenerative and neuropsychiatric disorders.

### Clinical implications

We found that both the cToM and eToM conditions of the CEPT could discriminate between neurologically healthy individuals and patients in the early phase of neurodegenerative disease. Persons with AD and bvFTD displayed more significant impairments in recognizing others’ beliefs on the cToM, which aligns with previous studies evaluating the theory of mind in neurodegeneration.^16,27,68^ Both persons with svPPA and PSP displayed somewhat less severely reduced capacity for cognitive perspective-taking, similar to previous reports,^15^ and persons with nfvPPA performed at the same level as controls.

The eToM condition of the CEPT detected impairments in persons with AD, bvFTD, svPPA, and PSP relative to controls, whereas nfvPPA displayed minor changes. While deficits in what has been termed “emotional Theory of Mind” have previously been seen in various neurodegenerative syndromes,^55,56,69–71^ other studies have almost exclusively used stimuli that require spontaneous emotion identification as a part of the test. However, the eToM condition of the CEPT does not require explicit emotion identification. Thus, our result shows for the first time that the ToM deficits seen in these patient groups are not simply due to difficulties with emotion reading but stem from impairments in cognitive processes directly related to perspective-taking, regardless of the content of the target individual's knowledge (i.e., location of an object, or an emotional state).

Somewhat worse performances on the eToM than cToM condition across patient groups suggests that following others’ knowledge of an explicitly identified emotion is potentially more difficult than following knowledge of the location of an object, perhaps due to the comparative lack of concreteness of the emotion, or because more complex neural processes are required to maintain the cognitive representation of another's emotion during working memory manipulations. We also found that the capacity to accurately read emotions still strongly correlated with performance on both the cToM and eToM conditions. This may reflect the fact that many patients with perspective-taking deficits also have emotion reading deficits, despite our evidence in this study that they are clinically dissociable symptoms.

The fact that both conditions of the CEPT showed strong correlations with tests in several other cognitive domains also has important clinical implications. Rather than a purer test that detects focal changes in a single functioning domain, the perspective-taking capacities measured by the CEPT depend on executive functioning, memory, visuospatial skills, attention, and some aspects of language. Thus, performance will be perturbed by alterations in any of those cognitive domains. As a result, low scores on this task during neuropsychological evaluations will not necessarily yield clear data on brain-behavior relationships or even specific diagnostic syndrome classification; however, reduced scores will be more likely to correspond to realistic deficits in social perspective-taking.

We found that among patients with neurodegenerative disease, performance on the cToM and eToM conditions of the CEPT could predict informant-reported neuropsychiatric symptoms and socioemotional sensitivity in real life; while other ToM tests used in persons with dementia may also correspond to real-life social deficits, studies often do not include this explicit comparison to allow this to be evaluated.^16,27^ Face-to-face tests that are able to reflect socioemotional and behavioral deficits in a more ecologically realistic manner are more likely to be of practical benefit to practitioners in both clinical research and clinical settings, particularly those where informant reports are unavailable. Rather than being a purely academic measure of perspective-taking, performance on the CEPT seems to reflect real-life socioemotional changes, even considering the early stage of the patients in our study.

In patients like those with bvFTD for whom the earliest neurologic signs of disease are not cognitive, but fall in the domain of social cognition, tests that can detect and quantify such impairments may be a pivotal part of diagnostic evaluation and disease staging.^42,72^ While the patient may not have insight into any changes, and close informants may not have the language to describe the changes they are perceiving (e.g., “She is more distant; her personality has changed.”), neuropsychological testing using validated social cognition tests like the CEPT can yield quantitative corroboration for the clinician or researcher.^73^ The role of these tests in early detection of neurodegenerative disease will become increasingly urgent as disease-modifying treatments for tau and TDP pathologies become publicly available.

### Limitations and conclusions

One limitation of this study is that it was performed in a population of predominantly white, well-educated individuals from the US, and we do not yet have evidence of the utility or generalizability of these results to more ethnically, culturally, or socioeconomically diverse samples. Further validation is needed to expand on normative and clinical data in these broader samples and determine if specific test materials or scoring adaptations are required for valid testing outside our reference sample. Also, though we were able to show good test-retest reliability and test stability in healthy individuals with the cToM condition, these psychometric features have not yet been evaluated in the eToM condition, which was constructed after considerable data collection with the cToM had already been conducted. Further studies are needed to determine the longitudinal characteristics of CEPT performance among patients throughout disease progression and whether the test remains useful at later stages. Analysis of the collinearity between CEPT scores and tests of executive function and emotion reading showed that both the cToM and eToM conditions corresponded to cognitive and emotional deficits in the patients. This may have been an artifact of the manner in which cognitive deficits co-occurred in our patient sample (e.g., patients with perspective taking deficits almost always showed concurrent emotion reading deficits because the disease causes stereotypical patterns of concurrent neural damage to distinct circuits), though this finding suggests further studies disambiguating the cToM and eToM conditions are needed. In particular, examining the specific neuroanatomic correlates of performance on each condition would be useful, and further would provide valuable data supporting the clinical interpretation of results during neuropsychological evaluations, by providing evidence for how test performance reflects the function of specific brain circuits affected by early neurodegeneration. For any neuropsychological test, psychometric precision in a face-to-face testing scenario does not necessarily indicate that the test will reliably or accurately reflect an individual's capacities in diverse and complex real-life situations, and the CEPT is no exception. Despite our comparatively small sample size, however, this study showed that the CEPT differentiates between healthy controls and participants in the early stages of neurodegenerative disease, and that test performance had a meaningful relationship with patients’ real-life behavior, including alterations in socioemotional and neuropsychiatric functioning. Given these useful characteristics, the CEPT may be a helpful addition to neuropsychological batteries attempting better to characterize social cognition in patients with early neurodegenerative disease.

## Supplemental Material

sj-docx-1-alz-10.1177_13872877251317683 - Supplemental material for Validation of the Cognitive-Emotional Perspective Taking test in patients with neurodegeneration Supplemental material, sj-docx-1-alz-10.1177_13872877251317683 for Validation of the Cognitive-Emotional Perspective Taking test in patients with neurodegeneration by Jonathan Adrián Zegarra-Valdivia, Tal Shany-Ur, Myrthe Gwen Rijpma, Patrick Callahan, Pardis Poorzand, Scott Grossman, Bailey McEachen, Joel H Kramer, Bruce L Miller and Katherine P Rankin in Journal of Alzheimer's Disease
