# Supplementary material for: Validation of the Cognitive-Emotional Perspective Taking test in patients with neurodegeneration
Source: J Alzheimers Dis. 2025 Mar 3;104(2):436–51. doi: 10.1177/13872877251317683 (PMC12231792; doi:10.1177/13872877251317683)
Supplement: sj-docx-1-alz-10.1177_13872877251317683 - Supplemental material for Validation of the Cognitive-Emotional Perspective Taking test in patients with neurodegeneration [file sj-docx-1-alz-10.1177_13872877251317683.docx]

**Supplemental Material**

**Validation of the Cognitive-Emotional Perspective Taking (CEPT) test in patients with neurodegeneration**

**Supplemental Table 1.** Psychometric properties of cognitive and emotional Theory of Mind test in the control group.

| Cronbach’s alpha | | | | | | |
| --- | --- | --- | --- | --- | --- | --- |
| Groups | **cToM** | **95% IC** | | **eToM** | **95% IC** | |
|  |  | **Lower limit** | **Upper limit** |  | **Lower limit** | **Upper limit** |
| Control group | 0.85 | 0.773 | 0.910 | 0.816 | 0.720 | 0.889 |
| Test-retest Reliability, two weeks (n=60) | | | | | | |
| ToM scores | **1st visit** | | **2nd visit** | | **Statistics** | |
|  | **Mean** | **SEM** | **Mean** | **SEM** | **t-test** | **p** |
| Total No Cheat | 11.9 | 0.045 | 11.92 | 0.036 | 0.148 | 0.883 |
| Total Cheat | 11.77 | 0.068 | 11.8 | 0.066 |  |  |
| Total Correct cToM* | 15.69 | 0.076 | 15.77 | 0.055 |  |  |
| Test-retest Reliability, one year (n=57) | | | | | | |
| ToM scores | **1st visit** | | **2nd visit** | | **Statistics** | |
|  | **Mean** | **SEM** | **Mean** | **SEM** | **t-test** | **p** |
| Total No Cheat | 11.83 | 0.069 | 11.87 | 0.059 | 0.0814 | 0.419 |
| Total Cheat | 11.81 | 0.06 | 11.8 | 0.089 |  |  |
| Total Correct cToM* | 15.67 | 0.088 | 15.74 | 0.084 |  |  |
| Spearman’s Correlation (Rho) | | | | | | |
| ToM test | | **cToM** | **eToM** | **CDR** | **Stroop** | **DART** |
| cToM | **ρ** | 1 | 0,136 | -.506** | 0.094 | 0.391 |
|  | **p** | . | 0,398 | 0.003 | 0.597 | 0.15 |
| eToM | **ρ** | 0.136 | 1 | -0.267 | 0.303 | 0.283 |
|  | **p** | 0.398 | . | 0.133 | 0.081 | 0.306 |

^#^p<0.10; **p<*0.05; and ***p<*0.01

**Supplemental Table 2.** Criterion-related validity, analyzing concurrent, convergent, and divergent validity in the clinic group and the total cohort.

| Spearman’s Correlation (Rho) | | | cToM | eToM | CDR | Stroop | | ER | Verbal |
| --- | --- | --- | --- | --- | --- | --- | --- | --- | --- |
| Control  group | **cToM** | **Rho** | 1 | 0.136 | -0.506** | 0.094 | | 0.199 | 0.473** |
|  |  | **p** | . | 0.398 | 0.003 | 0.597 | | 0.231 | 0.005 |
|  | **eToM** | **Rho** | 0.136 | 1 | -0.267 | 0.303 | | 0.315 | 0.119 |
|  |  | **p** | 0.398 | . | 0.133 | 0.081 | | 0.054 | 0.509 |
| Clinic  group | **cToM** | **Rho** | 1 | 0.646** | -0.424** | 0.441** | | 0.413** | 0.046 |
|  |  | **p** | . | 0.000 | 0.000 | 0.000 | | 0.000 | 0.474 |
|  | **eToM** | **Rho** | 0.646** | 1 | -0.447** | 0.304** | | 0.440** | 0.021 |
|  |  | **p** | 0.000 | . | 0.000 | 0.000 | | 0.000 | 0.737 |
| Total  group | **cToM** | **Rho** | 1 | 0.671** | -0.517** | 0.503** | | 0.471** | 0.164** |
|  |  | **p** | . | 0.000 | 0.000 | 0.000 | | 0.000 | 0.006 |
|  | **eToM** | **Rho** | 0.671** | 1 | -0.534** | 0.407** | | 0.500** | 0.131* |
|  |  | **p** | 0.000 | . | 0.000 | 0.000 | | 0.000 | 0.028 |
| Cronbach’s alpha | | | | | | | | | |
|  | **cToM (95% IC)** | | |  | | | **eToM (95% IC)** | | |
|  | ***α*** | **Lower limit** | **Upper limit** | ***α*** | **Lower limit** | | | **Upper limit** | |
| Clinic group | 0.927 | 0.913 | 0.939 | 0.910 | 0.893 | | | 0.926 | |
| Total group | 0.928 | 0.916 | 0.94 | 0.913 | 0.898 | | | 0.927 | |

*^#^p<*0.10; **p<*0.05; and ***p<*0.01

**Supplemental Table 3.** Subscale-test correlations in the control cohort for the CEPT.

| Subscale-Test Correlation | Spearman’s Correlation (Rho) | |
| --- | --- | --- |
| Item | cToM | eToM |
| No Cheat Control | 0.214** | 0.079 |
| Total: 1st Order + 2nd Order | 0.502** | *a* |
| No Cheat 1st Order | 0.708** | 0.608** |
| No Cheat 2nd Order | 0.168* | 0.169 |
| Cheat Control | 0.572** | 0.270** |
| Cheat 1st Order | 0.658** | 0.791** |
| Cheat 2nd Order | 0.252** | 0.149 |
| Total: Control | 0.734** | 0.270** |
| Total: 1st Order | 0.843** | 1.000** |
| Total: 2nd Order | 0.741** | 0.574** |
| Total: No Cheat | 0.778** | 0.792** |
| Total: Cheat | 0.963** | 0.984** |

*a=* no variance; **p<*0.05; and ***p<*0.01

**Supplemental Figure 1.** Bland Altman Test-retest stability in cToM

**A B**

**
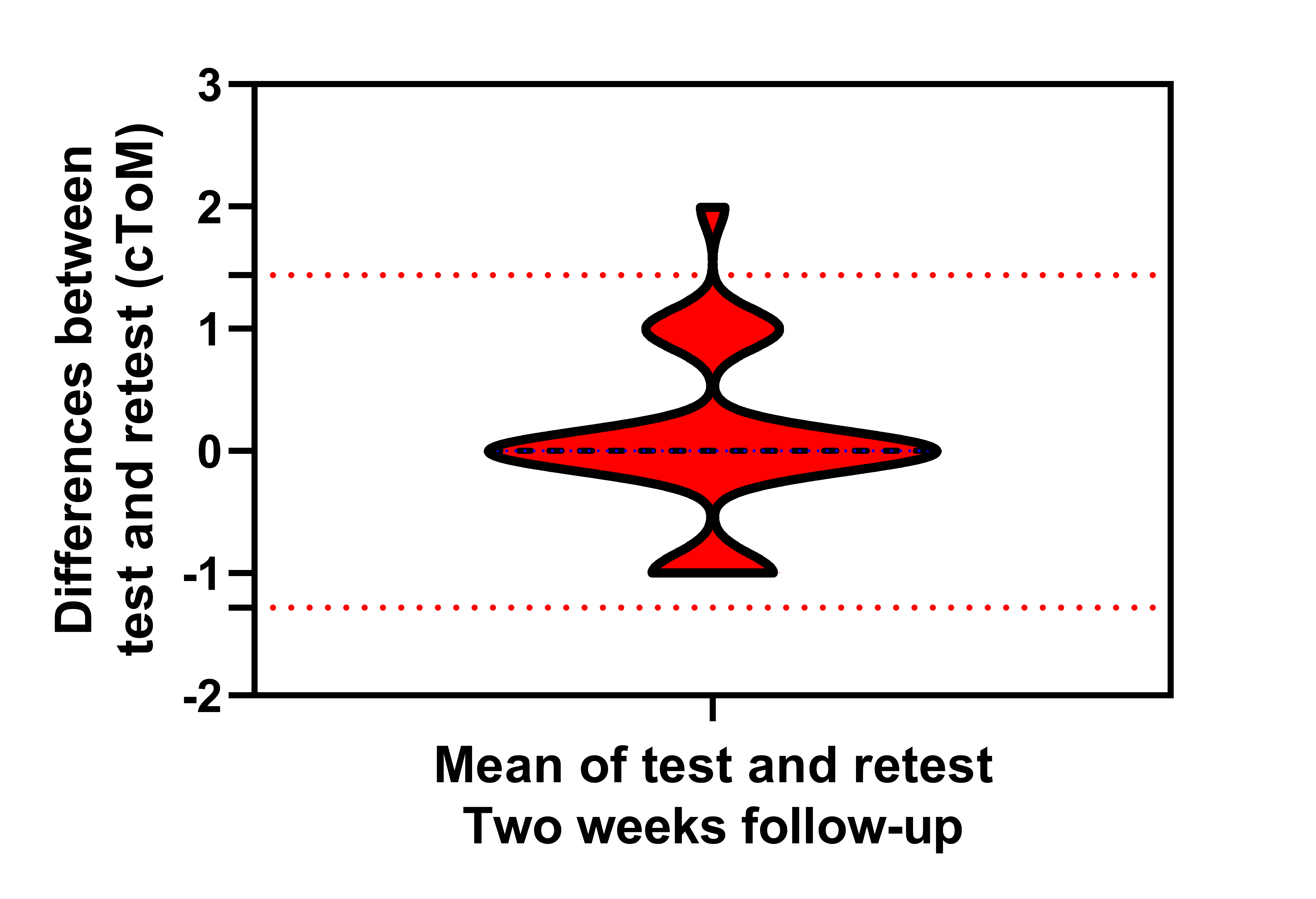

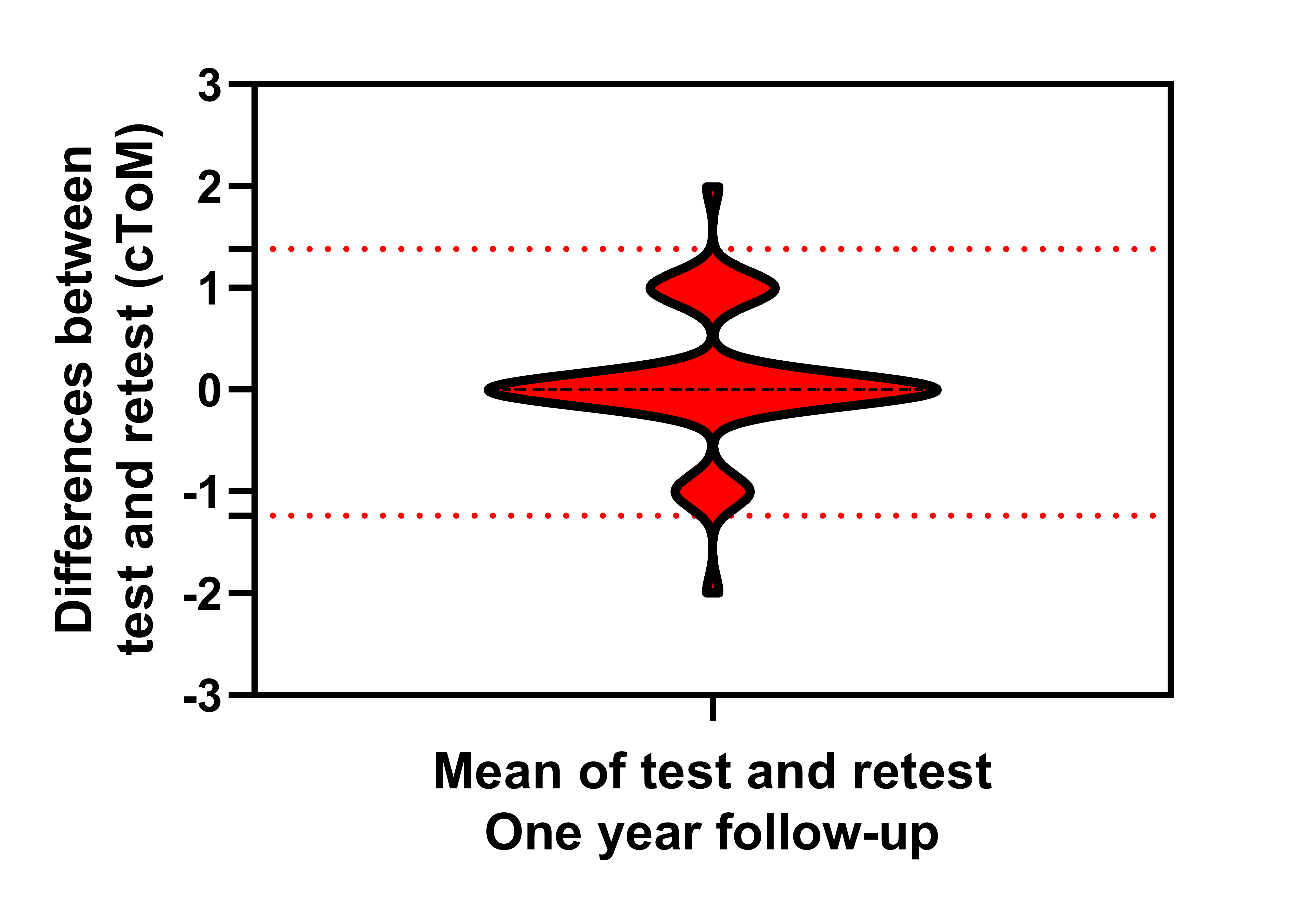
**

(A) cToM 2-week test-retest reliability; (B) cToM one-year test stability.

**Supplemental Table 4.** Multiple Regression model analyzing the contribution of cognitive and emotional functioning on cToM and eToM performance.

| cToM | B | 95% CI | | | Bootstrap | | SE B | | *β* | | *R^2^* | | *∆R^2^* |
| --- | --- | --- | --- | --- | --- | --- | --- | --- | --- | --- | --- | --- | --- |
|  |  | **LL** | **UL** |  | |  | |  | |  | |  | |
| Model |  |  |  |  | |  | |  | | 0.464 | | 0.449** | |
| Constant | 2.337 | -1.108 | 6.195 | 0.176 | | 1.845 | |  | |  |  |  |  |
| Age | 0.03 | -0.006 | 0.063 | -0.001 | | 0.018 | | 0.098 | |  |  |  |  |
| Sex | -0.019 | -0.623 | 0.585 | 0.001 | | 0.306 | | -0.003 | |  |  |  |  |
| Emotion Reading | 0.287** | 0.154 | 0.428 | -0.002 | | 0.07 | | 0.252** | |  |  |  |  |
| Stroop (interference Correct) | 0.055** | 0.035 | 0.075 | 0.001 | | 0.01 | | 0.317** | |  |  |  |  |
| Benson Copy | 0.193** | 0.032 | 0.325 | -0.004 | | 0.074 | | 0.148** | |  |  |  |  |
| Benson 10’ Delay | 0.225** | 0.127 | 0.316 | -0.002 | | 0.048 | | 0.316** | |  |  |  |  |
| eToM |  | | | | | | | | | | | | |
| Model |  | | | | | | | | | | 0.540 | | 0.529** |
| Constant | 5.349* | 3.009 | 7.673 | 0.023 | | 1.196 | |  | |  | |  | |
| Age | -0.014 | -0.036 | 0.009 | 0.001 | | 0.012 | | -0.054 | |  |  |  |  |
| Sex | 0.034 | -0.431 | 0.477 | -0.006 | | 0.233 | | 0.006 | |  |  |  |  |
| Emotion Reading | 0.316** | 0.208 | 0.438 | 0.001 | | 0.059 | | 0.351** | |  |  |  |  |
| Benson Copy | 0.155** | 0.064 | 0.259 | 0.001 | | 0.049 | | 0.174** | |  |  |  |  |
| CVLT (Total trials correct) | 0.109** | 0.064 | 0.15 | -0.001 | | 0.022 | | 0.292** | |  |  |  |  |
| Design Fluency (# correct) | 0.118** | 0.046 | 0.195 | 0.0001 | | 0.039 | | 0.174** | |  |  |  |  |

*^#^p<*0.10; **p<*0.05; ***p<*0.001
